# Serum multi-element profiling and a Metallobalance index for noninvasive differentiation of canine prostatic adenocarcinoma: a pilot case-control study

**DOI:** 10.3389/fvets.2026.1882881

**Published:** 2026-08-12

**Authors:** Naoki Yamada, Kumiko Ishigaki, Takeshi Hayakawa, Kaito Iida, Shoko Yamaoka, Pinhui Wang, Kazushi Asano

**Affiliations:** Laboratory of Veterinary Surgery, Department of Veterinary Medicine, College of Bioresource Sciences, Nihon University, Fujisawa, Kanagawa, Japan

**Keywords:** benign prostatic hyperplasia, biomarker, discriminant analysis, dog, multi-element, prostatic adenocarcinoma, serum

## Abstract

**Background:**

Noninvasive biomarkers for canine prostatic adenocarcinoma (PAC) are lacking. Serum multi-element profiling by inductively coupled plasma-mass spectrometry (ICP-MS) may provide diagnostic information.

**Objectives:**

To derive preliminary reference intervals (PRIs) for 18 serum elements in clinically healthy male dogs, assess a Metallobalance index (MBI) for distinguishing PAC from controls, and explore associations with lymph node metastasis, surgical margin status, and postoperative change.

**Animals:**

The study included 115 dogs: clinically healthy males (*n* = 65), dogs with benign prostatic hyperplasia (BPH; *n* = 25), one dog with a prostatic cyst, and dogs with histopathologically confirmed PAC (*n* = 24). The diagnostic control group comprised 91 dogs.

**Methods:**

Serum concentrations of 18 elements were measured by ICP-MS, and central 95% PRIs were derived. An 18-element linear discriminant analysis (LDA) model generated the MBI. Cutoffs maximized Youden’s index; apparent performance was summarized by sensitivity, specificity, accuracy, and area under the receiver operating characteristic curve (AUC). *Post hoc* analyses restricted controls to dogs aged ≥7 years and compared BPH with PAC using the Study 1 MBI equation without refitting. Element-specific and clinicopathological subgroup analyses were exploratory.

**Results:**

PRIs were derived for all elements. The MBI showed high apparent discrimination between PAC and controls (AUC, 0.983). At a cutoff of 0.8030, sensitivity, specificity, and accuracy were 100.0, 92.3, and 93.9%, respectively. Apparent AUCs were 0.984 with age-restricted controls and 0.998 for BPH versus PAC. As these were unvalidated training-set estimates, they should not be interpreted as clinical performance. In dogs with PAC, preoperative MBI was higher with lymph node metastasis, but did not differ by surgical margin status or clearly change 2 weeks postoperatively.

**Conclusions and clinical relevance:**

ICP-MS-based multi-element profiling with LDA showed high apparent training-set discrimination of canine PAC from controls, including benign prostatic disease. The association with lymph node metastasis warrants further study, whereas findings did not support short-term postoperative monitoring or a validated prognostic role. Independent validation in a larger prospective cohort is required before clinical application.

## Introduction

Trace elements and macrominerals underpin enzymatic catalysis, redox homeostasis, immune function, and tissue repair. Imbalance through deficiency or excess disrupts normal physiology and contributes to disease; examples include zinc-responsive dermatosis (1), iron-deficiency anemia (2), and copper-associated chronic hepatitis (3) in dogs.

In oncology, altered elemental profiles have been documented in both dogs and humans. Dogs with malignant disease have been reported to have lower serum selenium concentrations (4), and canine hepatocellular carcinoma tissue can show increased copper with decreased zinc (5). In human prostate cancer, higher serum concentrations of Na, Ca, Fe, Co, Cu, As, and Se together with lower Rb and Cs have been reported (6).

Traditional techniques such as atomic absorption spectrometry and inductively coupled plasma-optical emission spectrometry are susceptible to matrix effects and have limited multi-element capacity (7), whereas ICP-MS affords high sensitivity and simultaneous multi-element quantitation (8). Nevertheless, few veterinary studies have established serum PRIs for dogs using ICP-MS; one report provides PRIs for a subset of elements (9), and comparative control data exist for trace nutrients in other canine cohorts (10).

The MBI is a scalar multivariable discriminant score that summarizes the joint pattern of multiple serum elemental concentrations rather than interpreting each element as an independent biomarker. Related human studies have evaluated multi-element Metallobalance approaches primarily for cancer-risk discrimination (6, 11). Although such a score may integrate tumor-related, metabolic, inflammatory, nutritional, and organ-function effects on elemental homeostasis, the available literature does not establish that it predicts disease severity, prognosis, or treatment response. These potential applications therefore require prospective longitudinal validation.

Canine prostatic neoplasms include adenocarcinoma, urothelial carcinoma involving the prostatic urethra, and squamous cell carcinoma, with adenocarcinoma being the most common (12, 13). The disease is characterized by frequent local invasion and distant metastasis and shows poor outcomes under medical management (14); early detection followed by surgical intervention may improve quality of life and survival in selected cases (15), whereas medically treated cohorts still have limited survival (16). Unlike in humans, where prostate-specific antigen aids detection (17), there is no validated serum biomarker in dogs. BRAF mutation testing is emerging but has limited sensitivity for PAC (18, 19).

The objectives of this study were to establish ICP-MS-based serum PRIs for 18 elements in clinically healthy male dogs and to evaluate whether an LDA-based multi-element MBI could differentiate canine PAC from controls, including benign prostatic disease. Exploratory objectives were to assess associations with lymph node metastasis, surgical margin status, and short-term postoperative change.

## Materials and methods

### Study design and animals

A total of 115 dogs were included. Clinically healthy male dogs (*n* = 65) undergoing wellness examinations at participating facilities between June 2017 and August 2023 were enrolled for PRI derivation. Dogs were considered clinically healthy when no clinical signs of illness were reported and no clinically relevant abnormalities were identified on physical examination, complete blood count, or serum biochemical testing. Dogs with a known history of, or clinical or clinicopathological findings suggestive of, renal disease, hepatic disease, or systemic inflammatory disease were excluded. Urinalysis was not performed uniformly and therefore was not used as an eligibility criterion.

**Figure 1:**
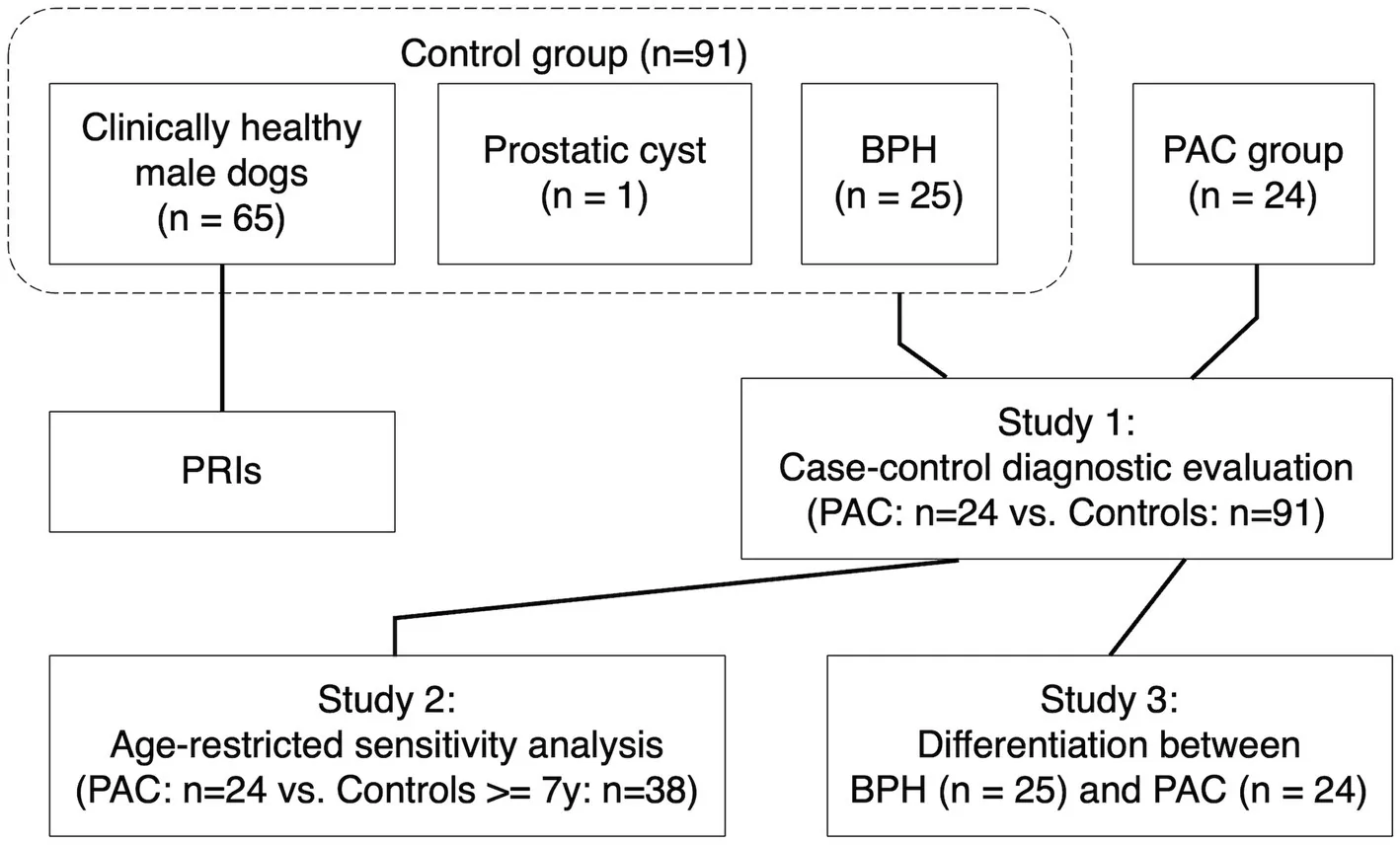
Flow diagram of the study population and analyses. The control group comprised 65 clinically healthy male dogs, 25 dogs with benign prostatic hyperplasia, and one dog with a prostatic cyst. Study 2 was a *post hoc* age-restricted sensitivity analysis and not an individually matched or independent validation analysis. BPH, benign prostatic hyperplasia; PAC, prostatic adenocarcinoma; PRI, preliminary reference interval.

The diagnostic control group comprised the clinically healthy cohort (*n* = 65), dogs with BPH (*n* = 25), and one dog with a prostatic cyst, yielding 91 controls. No dogs with prostatitis or other prostatic neoplasms were included in the control group. Dogs with histopathologically confirmed PAC that underwent surgical excision at our hospital between October 2012 and August 2021 formed the PAC group (*n* = 24). Enlarged regional lymph nodes identified by imaging or gross inspection were excised for histopathological examination when clinically indicated. The study groups and analyses are summarized in Figure 1.

### Diagnostic criteria

BPH was defined when at least one of the following criteria was met: (i) prostatic width exceeding 50% of pelvic inlet width on ventrodorsal radiography (20); (ii) prostatic long-axis length exceeding 70% of the pubis-to-sacrum distance on lateral radiography (21); or (iii) ultrasonographically measured prostatic volume (length × width × height × 0.523) exceeding the predicted maximum volume calculated as 0.867 × body weight + 1.885 × age + 15.88 (22).

**Figure 2:**
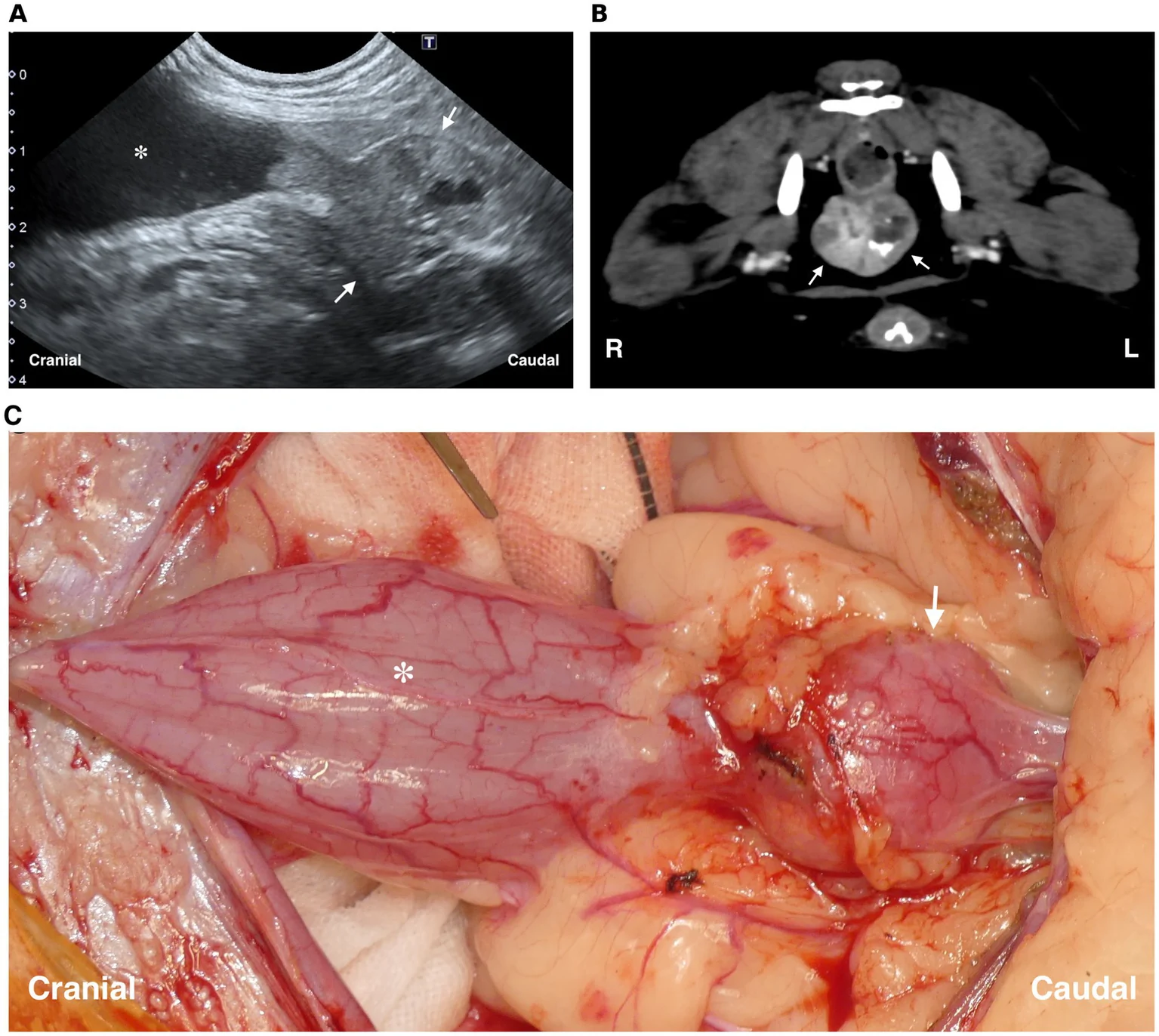
Representative preoperative imaging and intraoperative findings in a dog with histopathologically confirmed prostatic adenocarcinoma. **(A)** Sagittal B-mode ultrasonographic image. The asterisk indicates the urinary bladder, and white arrows indicate the heterogeneously echogenic prostatic lesion with cysts. **(B)** Transverse contrast-enhanced computed tomographic image. White arrows indicate the enlarged, heterogeneously enhancing prostatic lesion with partial calcification; R and L denote the right and left sides, respectively. **(C)** Intraoperative photograph obtained during prostatocystectomy in the same dog. The asterisk indicates the urinary bladder, and the white arrow indicates the prostate. The imaging findings are representative and were not used as stand-alone diagnostic criteria.

**Figure 3:**
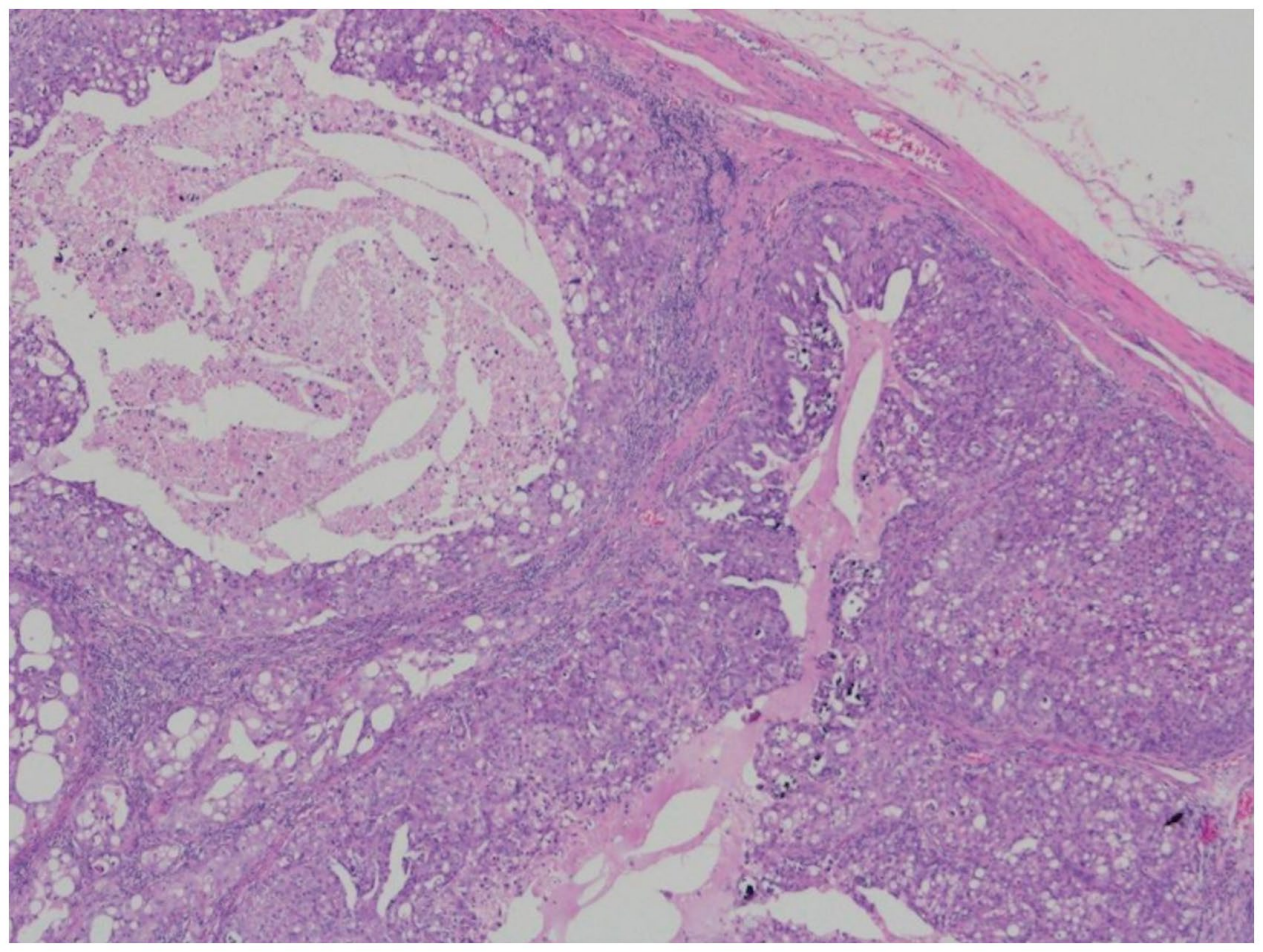
Representative low-magnification histopathological findings in the dog shown in Figure 2. A hematoxylin and eosin-stained section shows extensive effacement of the normal prostatic architecture by a neoplastic epithelial proliferation arranged in tubular and solid growth patterns. Histopathological examination of the resected specimen confirmed prostatic adenocarcinoma.

PAC was confirmed histopathologically following prostatectomy or prostatocystectomy. Representative preoperative B-mode ultrasonographic and contrast-enhanced computed tomographic images and an intraoperative photograph from the same histopathologically confirmed case are shown in Figures 2A–C, respectively. A representative low-magnification histopathological section from the same dog is shown in Figure 3. These imaging findings are presented for illustrative purposes and were not used as stand-alone diagnostic criteria for inclusion in the PAC group.

Resected sublumbar lymph nodes were examined histopathologically when lymphadenectomy was performed. Surgical margins were also evaluated histopathologically. Histologically complete excision with negative margins was classified as R0 resection, whereas microscopic tumor at a surgical margin was classified as R1 resection.

### Sample collection and elemental analysis

Venous blood was collected, centrifuged at 3,000 rpm for 10 min, and the separated serum was stored at −80 °C until analysis. Serum samples (50 μL) were transferred to precleaned polypropylene tubes. For digestion, 125 μL of 61% nitric acid and 25 μL of 30% hydrogen peroxide were added, and the samples were heated at 70 °C for 16 h. After cooling to room temperature, Be, Y, Te, and Rh were added as internal standards at nominal concentrations of 50, 5, 50, and 1 μg/L, respectively. The digests were diluted to a final volume of 2.5 mL with ultrapure water. Samples exceeding the highest calibration standard were further diluted and reanalyzed, and the reported concentrations were corrected for the additional dilution. For surgical cases, serum sampling was repeated 2 weeks postoperatively.

Concentrations of 18 elements (Li, Na, Mg, P, S, K, Ca, V, Fe, Co, Cu, Zn, As, Se, Rb, Sr, Mo, and Cs) were quantified using an Agilent 7,800 ICP-MS equipped with an SPS 4 autosampler (Agilent Technologies, Santa Clara, CA, USA). Measurements were performed using no-gas, H₂, He, or high-energy He modes, as appropriate for each analyte. Element-specific gas-mode assignments and analytical performance metrics are provided in Table 1, and the operating conditions for each gas mode are provided in Table 2.

**Table 1:** Analytical performance metrics of the ICP-MS method for 18 elements.

Element	Unit	Gas mode	LOD	LOQ	Intra-assay CV (%)	Inter-assay CV (%)	Spike recovery (%)	Seronorm recovery (%)
Li	μg/L	No gas	0.0030	0.0139	1.53	5.14	99.7	95.9
Na	mg/L	He	0.0034	0.0161	1.52	2.06	106.8	97.0
Mg	mg/L	He	0.0001	0.0004	1.76	2.52	105.9	94.1
P	mg/L	He	0.0009	0.0044	1.43	2.58	100.2	83.6
S	mg/L	HEHe	0.0514	0.2421	1.46	2.22	112.5	95.3
K	mg/L	H₂	0.0006	0.0027	1.46	1.97	111.8	97.0
Ca	mg/L	H₂	0.0021	0.0100	1.43	2.29	98.6	98.8
V	μg/L	He	0.0003	0.0015	3.90	5.22	111.3	100.3
Fe	μg/L	H₂	0.1674	0.7890	3.69	7.68	92.4	87.5
Co	μg/L	He	0.0006	0.0030	2.28	3.70	99.6	88.7
Cu	μg/L	He	0.0101	0.0475	1.34	3.87	99.2	95.6
Zn	μg/L	He	0.1051	0.4957	1.57	2.72	96.6	95.7
As	μg/L	He	0.0006	0.0028	12.70	13.40	108.2	90.6
Se	μg/L	H₂	0.0023	0.0110	2.21	2.83	92	100.2
Rb	μg/L	He	0.0007	0.0035	2.79	2.90	101.5	98.9
Sr	μg/L	He	0.0032	0.0150	1.54	1.62	105.6	97.7
Mo	μg/L	He	0.0004	0.0019	3.40	5.24	110	89.6
Cs	μg/L	He	0.0006	0.0027	14.60	18.40	103.7	86.0

CV, coefficient of variation; LOD, limit of detection; LOQ, limit of quantification. LOD was calculated as 3 times the standard deviation of 10 procedural blanks. Because blank-corrected concentrations were used, LOQ was calculated as 14.1 times the standard deviation of the procedural blanks, using the multipliers specified in JIS K 0133:2007. Intra-assay CV was determined from 32 replicate measurements of a pooled serum sample within one analytical run. Inter-assay CV was calculated from repeated measurements of the pooled serum sample on separate analytical days. Spike recovery was assessed after addition of known amounts of elemental standards to serum samples. Seronorm recovery was calculated by comparing measured concentrations of Seronorm Trace Elements Serum L-1 with the corresponding lot-specific assigned values.

**Table 2:** ICP-MS operating conditions for different collision/reaction-cell gas modes.

Operating parameter	No gas	H₂	He	HEHe
RF power (W)	1,550	1,550	1,550	1,550
Plasma gas flow rate (L/min)	15	15	15	15
Auxiliary gas flow rate (L/min)	0.9	0.9	0.9	0.9
Nebulizer gas flow rate (L/min)	1.05	1.05	1.05	1.05
He cell-gas flow rate (mL/min)	—	—	4.3	10
H₂ cell-gas flow rate (mL/min)	—	6	—	—

RF, radiofrequency; H₂, hydrogen; He, helium; HEHe, high-energy helium mode; —, not applicable.

Quantitation was performed using multipoint external calibration. Calibration solutions for Li, V, Co, As, Se, Rb, Sr, Mo, and Cs were prepared from XSTC-622B multi-element standard solution (SPEX CertiPrep, Metuchen, NJ, USA). Calibration solutions for Na, Mg, S, K, Ca, Fe, Cu, and Zn were prepared from the corresponding single-element standards from Kanto Chemical Co., Inc., Japan: Sodium Standard Solution (Na 1000; catalog no. 37821-1B), Magnesium Standard Solution 1 (Mg 1000; 25840-2B), Sulfur Standard Solution (S 1000; 37377-96), Potassium Standard Solution (K 1000; 32832-2B), Calcium Standard Solution 1 (Ca 1000; 07998-2B), Iron Standard Solution (Fe 1000; 20247-2B), Copper Standard Solution (Cu 1000; 08046-2B), and Zinc Standard Solution (Zn 1000; 48096-2B). Phosphorus calibration solutions were prepared from Phosphorus Standard Solution (P 1000; certified reference material; catalog no. 160-29631; FUJIFILM Wako Pure Chemical Corporation, Japan).

The number of calibration levels, including a zero-concentration calibration blank, was four for Na, Mg, P, S, K, and Ca; five for V, Co, Cu, Zn, As, and Cs; six for Li, Fe, and Mo; seven for Rb and Sr; and eight for Se. Calibration ranges were 0–100 mg/L for Na, 0–1 mg/L for Mg, 0–5 mg/L for P, K, and Ca, and 0–50 mg/L for S. For elements reported in μg/L, the ranges were 0–0.3 μg/L for V, Co, As, and Cs; 0–2 μg/L for Li and Mo; 0–10 μg/L for Rb and Sr; 0–20 μg/L for Se; 0–50 μg/L for Cu and Zn; and 0–250 μg/L for Fe. Calibration curves were fitted using unweighted linear regression with a freely estimated intercept and were not forced through the origin. Curves were accepted when *R*^2^ was at least 0.9995; this criterion was met for every element.

Ten procedural blanks were prepared using ultrapure water in place of serum and were processed through the complete digestion and analytical procedure. Final concentrations were calculated after subtraction of the mean procedural-blank concentration and correction for the dilution factor. Analytical performance was assessed by spike-recovery testing, analysis of Seronorm Trace Elements Serum L-1 as a serum quality-control material with lot-specific assigned values, and intra- and inter-assay precision measurements. The LOD was calculated as 3 times the standard deviation of the procedural blanks, and the LOQ was calculated as 14.1 times the standard deviation because blank-corrected concentrations were used, using the multipliers specified in JIS K 0133:2007. Element-specific LODs, LOQs, intra-assay CVs, inter-assay CVs, spike recoveries, and Seronorm recoveries are reported in Table 1.

All individual elemental concentrations were at or above the corresponding element-specific LOQs. Therefore, no measurements were below the LOD or between the LOD and LOQ, and no censoring, substitution, exclusion, or imputation was required.

### Preliminary reference intervals and statistical analysis

For clinically healthy dogs, PRIs were defined as the central 95% interval. Distributional characteristics of elemental concentrations and other continuous variables were inspected using histograms, Q–Q plots, and boxplots, and normality was assessed using the Shapiro–Wilk test. Parametric limits (mean ± 1.96 SD) or nonparametric limits (2.5th and 97.5th percentiles) were applied according to distributional characteristics and published recommendations for reference-interval estimation (23, 24). Approximately symmetric variables are presented as mean ± SD, whereas skewed variables are presented as median [interquartile range]. Age distributions in focused analyses were compared using the Mann–Whitney *U* test.

For the primary diagnostic analysis (Study 1), an LDA model was constructed using the full control group (*n* = 91) and the PAC group (*n* = 24). The scalar discriminant score generated from all 18 elements was defined as the MBI. The LDA model was prespecified as the primary multivariable analysis and was not based on variable selection from exploratory univariate comparisons. The same dataset was used for model derivation and performance estimation; therefore, all reported diagnostic metrics are apparent training-set estimates. No cross-validation, bootstrapping, or external validation was performed.

Two *post hoc* focused analyses were conducted. For the age-restricted sensitivity analysis (Study 2), the full control group was restricted to dogs aged 7 years or older (*n* = 38) and compared with the PAC group (*n* = 24). This restriction was intended to reduce the marked age imbalance in the primary comparison; it did not constitute individual matching or independent validation. For the BPH-versus-PAC analysis (Study 3), dogs with BPH (*n* = 25) were compared with dogs with PAC (*n* = 24). In both focused analyses, the Study 1 MBI equation and coefficients were applied without refitting.

Diagnostic performance was summarized using sensitivity, specificity, accuracy, and ROC analysis with AUC. Cutoff values were selected from the corresponding ROC curves by maximizing Youden’s index. Exploratory comparisons of individual elemental concentrations were evaluated using *p* values from Wilks’ Lambda tests within the discriminant analysis framework. Differences in the MBI based on lymph node metastasis and surgical margin status were assessed using the Mann–Whitney *U* test, while postoperative changes in the MBI were evaluated using the Wilcoxon signed-rank test. Individual elemental *p* values were not adjusted for multiplicity and were interpreted descriptively. Analyses were performed using BellCurve for Excel version 4.00 and GraphPad Prism version 6.0 for macOS.

## Results

### Study population and signalment

A total of 115 dogs were included: 65 clinically healthy male dogs, 25 dogs with BPH, one dog with a prostatic cyst, and 24 dogs with PAC. The control group therefore comprised 91 dogs. The clinically healthy cohort had a median age of 4.0 [2.0–8.0] years; 54 dogs were neutered and 11 were sexually intact. The most common breeds were mixed-breed dogs (*n* = 14), Chihuahuas (*n* = 7), Shiba Inus (*n* = 7), and Toy Poodles (*n* = 7). The PAC group had a median age of 11.5 [10.0–13.0] years; 22 dogs were neutered and 2 were sexually intact. The complete available signalment and breed distribution are summarized in Table 3.

**Table 3:** Signalment characteristics of clinically healthy male dogs and dogs with prostatic adenocarcinoma or benign prostatic disease.

Characteristic	Clinically healthy (*n* = 65)	PAC (*n* = 24)	BPD (*n* = 26)
Age (years)			
Median [IQR]	4.0 [2.0–8.0]	11.5 [10.0–13.0]	10.0 [7.0–13.0]
Mean ± SD	5.0 ± 3.6	11.4 ± 2.0	10.0 ± 3.5
Neuter status			
Neutered	54	22	—
Intact	11	2	26
Breeds			
Miniature Dachshund	2	11	4
Chihuahua	7	1	5
Pomeranian	3	2	6
Toy Poodle	7	—	1
Shiba Inu	7	—	—
Papillon	2	3	—
Labrador Retriever	3	1	—
Yorkshire Terrier	4	—	—
Beagle	1	2	—
Welsh Corgi	3	—	—
Miniature Schnauzer	1	1	—
Jack Russell Terrier	—	1	1
French Bulldog	2	—	—
Golden Retriever	2	—	—
Cavalier King Charles Spaniel	2	—	—
Pug	2	—	—
American Cocker Spaniel	1	—	1
Border Collie	1	—	—
Flat-Coated Retriever	1	—	—
Italian Greyhound	—	1	—
Great Dane	—	—	1
Mixed-breed	14	1	3
Breed not recorded	—	—	4

Data are presented as median [IQR], mean ± SD, or number of dogs, as indicated. The benign prostatic disease group comprised dogs with benign prostatic hyperplasia (*n* = 25) and one dog with a prostatic cyst (*n* = 1). BPD, benign prostatic disease; IQR, interquartile range; PAC, prostatic adenocarcinoma; SD, standard deviation; —, no dogs.

### Preliminary reference interval derivation

PRIs were derived for all 18 elements. Units were mg/L for Na, Mg, P, S, K, and Ca and μg/L for all other elements. Distributional assessments guided the use of parametric or nonparametric procedures. Summary statistics and lower and upper reference limits are reported in Table 4.

**Table 4:** Serum concentrations and preliminary reference intervals for 18 elements in clinically healthy male dogs (*n* = 65).

Element	Mean	Median	SD	Minimum	Maximum	Normally distributed (Shapiro–Wilk test)	PRI	
							LRL	URL
Li	2.34	1.65	2.86	0.65	21.8	No	0.73	14.35
Na	3,578	3,572	110.2	3,283	3,784	Yes	3,362	3,794
Mg	20.6	20.4	2.22	16.2	26.9	Yes	16	25
P	198	196	39.1	139.9	349.2	No	142	300
S	835	830	70.7	655.5	1,043	Yes	696.6	973.8
K	183.1	184.7	19.4	129	227.9	No	130.2	222.1
Ca	103.8	103.5	6.0	89.09	117.6	Yes	92.1	115.5
V	1.77	1.25	1.52	0.41	7.73	No	0.54	7.70
Fe	2,886	2,246	4,130	760	34,590	No	950	16,561
Co	0.77	0.30	1.62	0.10	11.83	No	0.10	7.40
Cu	433	428	70	292	594	Yes	296	570
Zn	747	705	181	454	1,204	No	482	1,194
As	3.0	1.23	4.15	0.32	22.33	No	0.36	20.05
Se	320	318	57	205.5	434.9	Yes	208	432
Rb	242	232	86	63.8	499	Yes	73	411
Sr	52	48	17	23.6	110	No	25	100
Mo	9.5	8.4	6.7	1.175	30.12	No	1.8	29.2
Cs	0.72	0.63	0.39	0.126	2.155	No	0.17	1.99

SD, standard deviation; PRI, preliminary reference interval; LRL, lower reference limit; URL, upper reference limit. Units are mg/L for Na, Mg, P, S, K, and Ca and μg/L for all other elements.

### Representative imaging and histopathological findings

Representative ultrasonographic, contrast-enhanced CT, intraoperative, and histopathological findings from the same dog with PAC are shown in Figures 2, 3. Histopathological examination of the representative case confirmed PAC. The resected prostate showed extensive replacement of the normal prostatic architecture by a neoplastic epithelial proliferation arranged in tubular and solid growth patterns.

### Study 1: Primary control-versus-PAC diagnostic analysis

Exploratory comparisons of the 18 individual elemental concentrations are summarized in Table 5. Na, Ca, Cu, Zn, and Se differed between controls and dogs with PAC, with Na, Ca, Cu, and Se higher in the PAC group. These comparisons were exploratory, were not adjusted for multiplicity, and were not used for feature selection.

**Table 5:** Exploratory comparison of serum elemental concentrations between control dogs and dogs with prostatic adenocarcinoma.

Element	Control (*n* = 91)	PAC (*n* = 24)	*p* value
Li	1.66 [1.17–2.40]	1.59 [0.97–2.31]	0.229
Na	3,523 [3,352–3,638]	3,668 [3,616–3,708]	<0.001
Mg	20.5 ± 2.1	20.5 ± 1.9	0.759
P	187 [164–217]	212 [173–235]	0.663
S	851 ± 76	866 ± 72	0.080
K	184.7 [175.0–194.9]	185.0 [174.0–191.8]	0.193
Ca	101.4 [97.3–106.3]	103.8 [99.8–110.1]	0.041
V	1.06 [0.69–1.75]	1.39 [1.11–2.07]	0.208
Fe	2,136 [1,716–2,833]	1,688 [1,249–2,282]	0.901
Co	0.24 [0.15–0.53]	0.20 [0.13–0.30]	0.465
Cu	463 [403–563]	544 [507–659]	< 0.001
Zn	677 [587–814]	698 [559–788]	0.033
As	1.10 [0.69–2.98]	1.15 [0.59–1.94]	0.984
Se	322 ± 53	370 ± 49	< 0.001
Rb	234 [193–302]	208 [179–245]	0.186
Sr	47 [39–61]	47 [40–72]	0.527
Mo	7.7 [3.5–12.8]	5.9 [4.2–10.0]	0.645
Cs	0.63 [0.48–0.99]	0.89 [0.55–1.04]	0.311

Data are presented as mean ± SD for approximately symmetric variables and median [IQR] for skewed variables. *p* values were obtained from Wilks’ Lambda tests in the discriminant-analysis framework. These comparisons were exploratory and were not adjusted for multiplicity. PAC, prostatic adenocarcinoma; SD, standard deviation; IQR, interquartile range. Units are mg/L for Na, Mg, P, S, K, and Ca and μg/L for all other elements.

**Figure 4:**
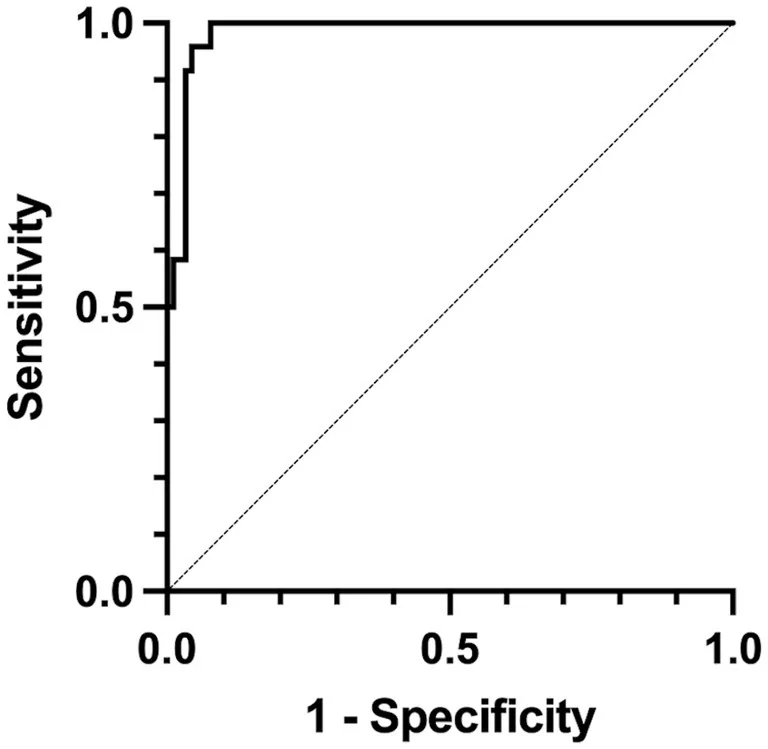
Receiver operating characteristic curve showing the apparent diagnostic performance of the Metallobalance index for differentiating prostatic adenocarcinoma from the full control group. The area under the curve was 0.983. At the Youden index-derived cutoff of 0.8030, sensitivity, specificity, and accuracy were 100.0, 92.3, and 93.9%, respectively. Because the same dataset was used for model derivation and performance estimation, these metrics represent apparent training-set performance and may overestimate performance in independent cohorts.

Using all 18 elements, the MBI showed high apparent discriminatory performance for PAC versus the full control group, with an AUC of 0.983. At the Youden-index-derived cutoff of 0.8030, sensitivity, specificity, and accuracy were 100.0, 92.3, and 93.9%, respectively (Figure 4). Because the same observations were used for model derivation and performance estimation, these metrics represent apparent training-set performance and may overestimate performance in independent cohorts.

### Study 2: Age-restricted sensitivity analysis

**Figure 5:**
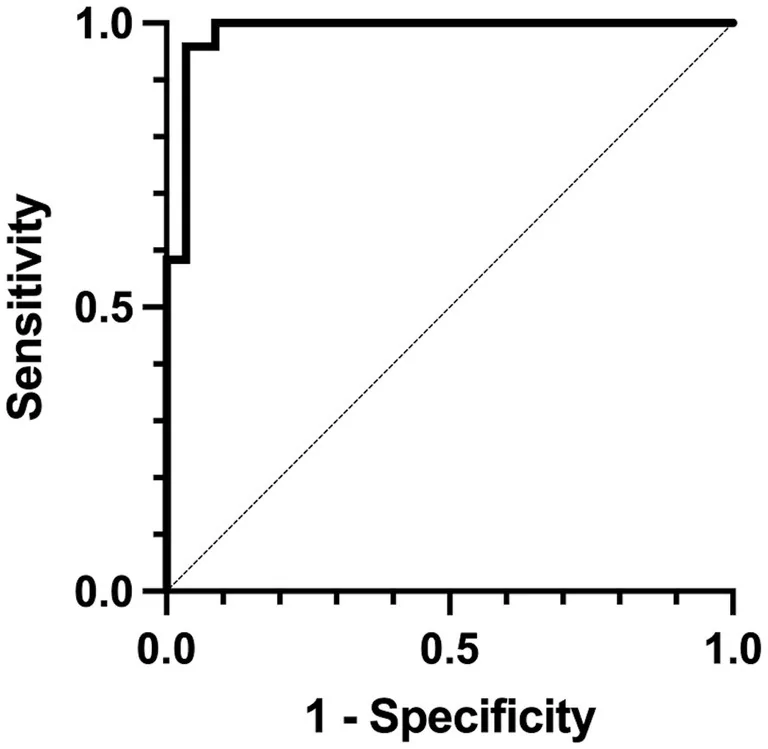
Receiver operating characteristic curve for the post hoc age-restricted sensitivity analysis comparing dogs with prostatic adenocarcinoma (*n* = 24) with controls aged 7 years or older (*n* = 38). The unchanged Study 1 Metallobalance index equation was applied without refitting, yielding an apparent area under the curve of 0.984. This subset analysis was not an individually matched design and did not constitute independent validation.

The *post hoc* age-restricted analysis included controls aged 7 years or older (*n* = 38; median age, 10.5 [8.0–13.0] years) and dogs with PAC (*n* = 24; median age, 11.5 [10.0–13.0] years). The age distributions were not significantly different by the Mann–Whitney *U* test (*p* = 0.1293), although this does not establish age equivalence. The unchanged Study 1 MBI equation yielded an apparent AUC of 0.984 (Figure 5). This focused analysis was performed in a subset of the model-development data and was not an independent validation analysis.

### Study 3: BPH-versus-PAC analysis

**Figure 6:**
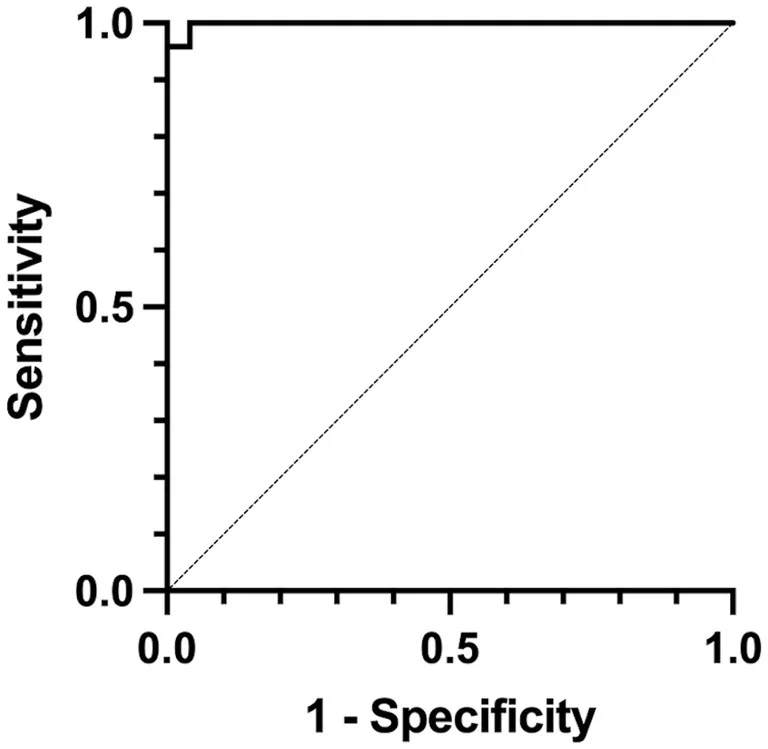
Receiver operating characteristic curve for differentiating benign prostatic hyperplasia from prostatic adenocarcinoma using the Metallobalance index equation derived in Study 1 without refitting. The area under the curve was 0.998. At the Youden-index-derived cutoff of 0.7895, sensitivity, specificity, and accuracy were 100.0, 96.0, and 98.0%, respectively. Because both groups contributed to model development, these values represent apparent training-set subgroup performance. BPH, benign prostatic hyperplasia; PAC, prostatic adenocarcinoma.

Dogs with BPH (*n* = 25; median age, 10.0 [7.0–13.0] years) and dogs with PAC (*n* = 24; median age, 11.5 [10.0–13.0] years) did not have significantly different age distributions (*p* = 0.2223). When the unchanged Study 1 MBI equation was applied without refitting, the apparent AUC was 0.998. At the Youden-index-derived cutoff of 0.7895, sensitivity, specificity, and accuracy were 100.0, 96.0, and 98.0%, respectively (Figure 6). Because both subgroups contributed to the model-development dataset, this was a training-set subgroup analysis rather than independent validation.

### Exploratory perioperative and pathological associations

**Figure 7:**
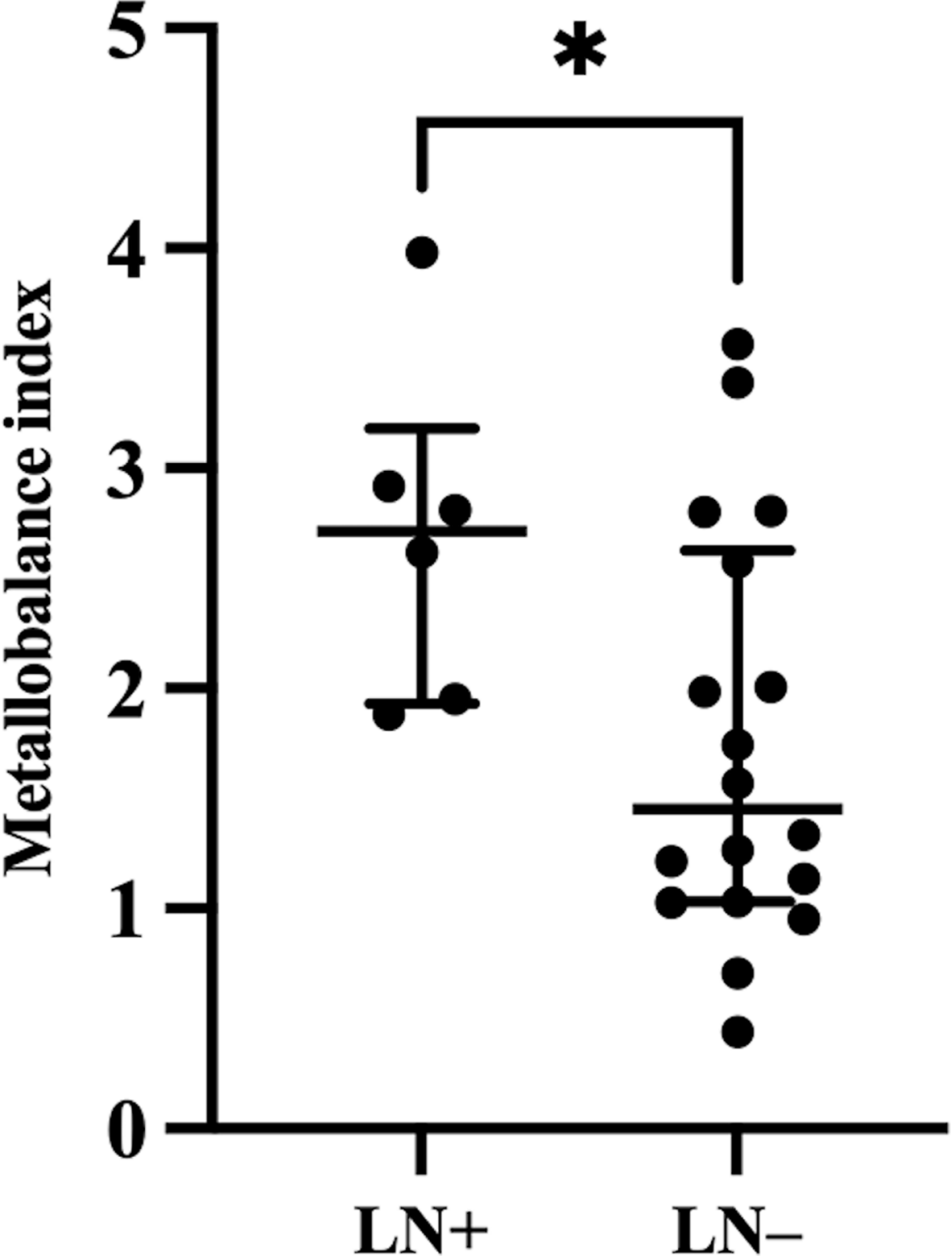
Metallobalance index according to lymph node metastasis status in dogs with prostatic adenocarcinoma. Values are presented as individual observations with median [IQR]. The index was higher in dogs with lymph node metastasis (LN+, 2.71 [1.93–3.18]) than in dogs without lymph node metastasis (LN−, 1.45 [1.03–2.63]; *p* = 0.0330). The *p* value was obtained using the Mann–Whitney *U* test (*p* = 0.0330), and the comparison was exploratory. ^*^p < 0.05. LN, lymph node; IQR, interquartile range.

**Figure 8:**
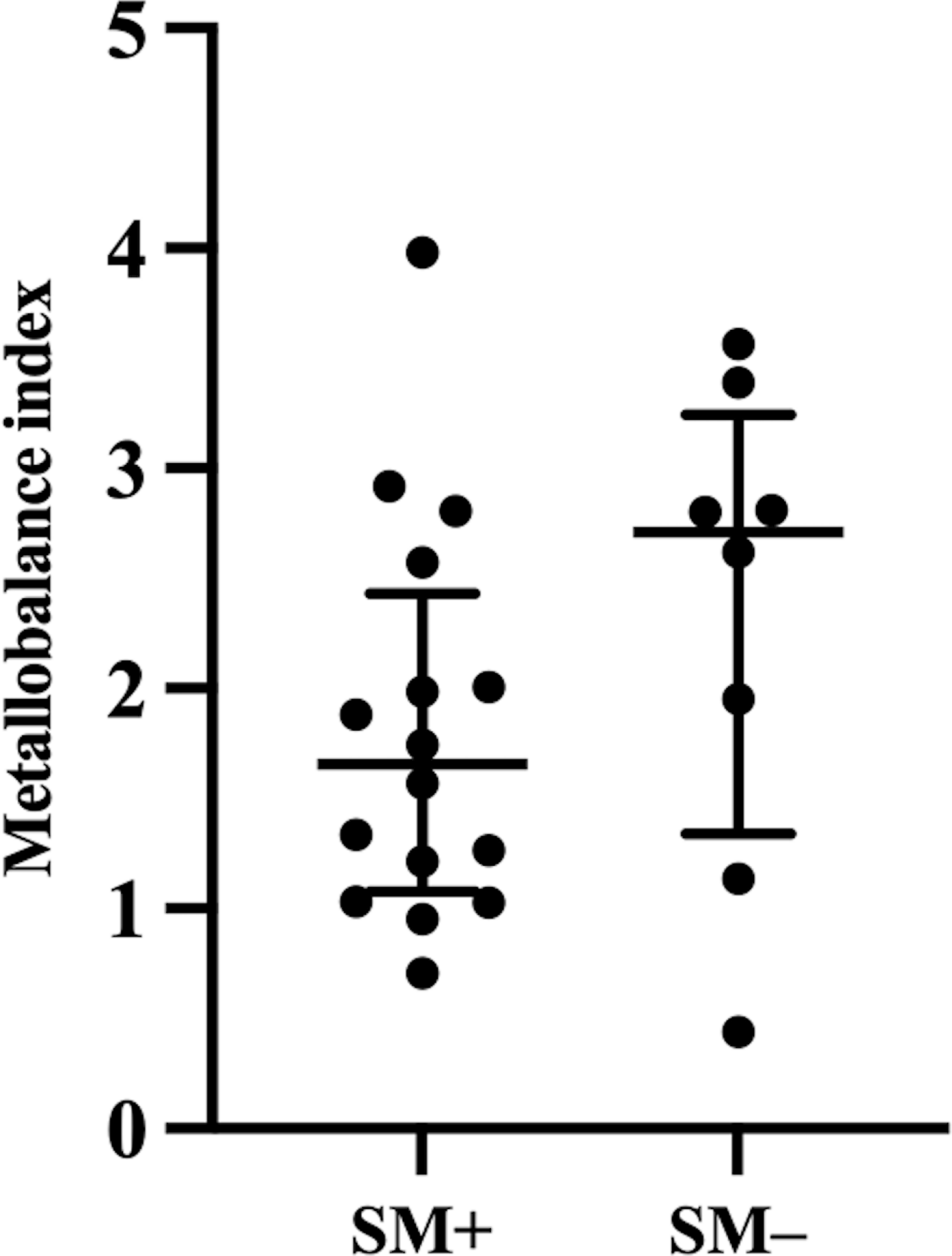
Metallobalance index according to surgical margin status in dogs with prostatic adenocarcinoma. Values are presented as individual observations with median [IQR]. No clear difference was observed between dogs with R1/positive surgical margins (SM+, 1.66 [1.10–2.43]) and dogs with R0/negative surgical margins (SM−, 2.71 [1.34–3.24]; *p* = 0.2381). The *p* value was obtained using the Mann–Whitney *U* test, and the comparison was exploratory. SM, surgical margin; IQR, interquartile range.

**Figure 9:**
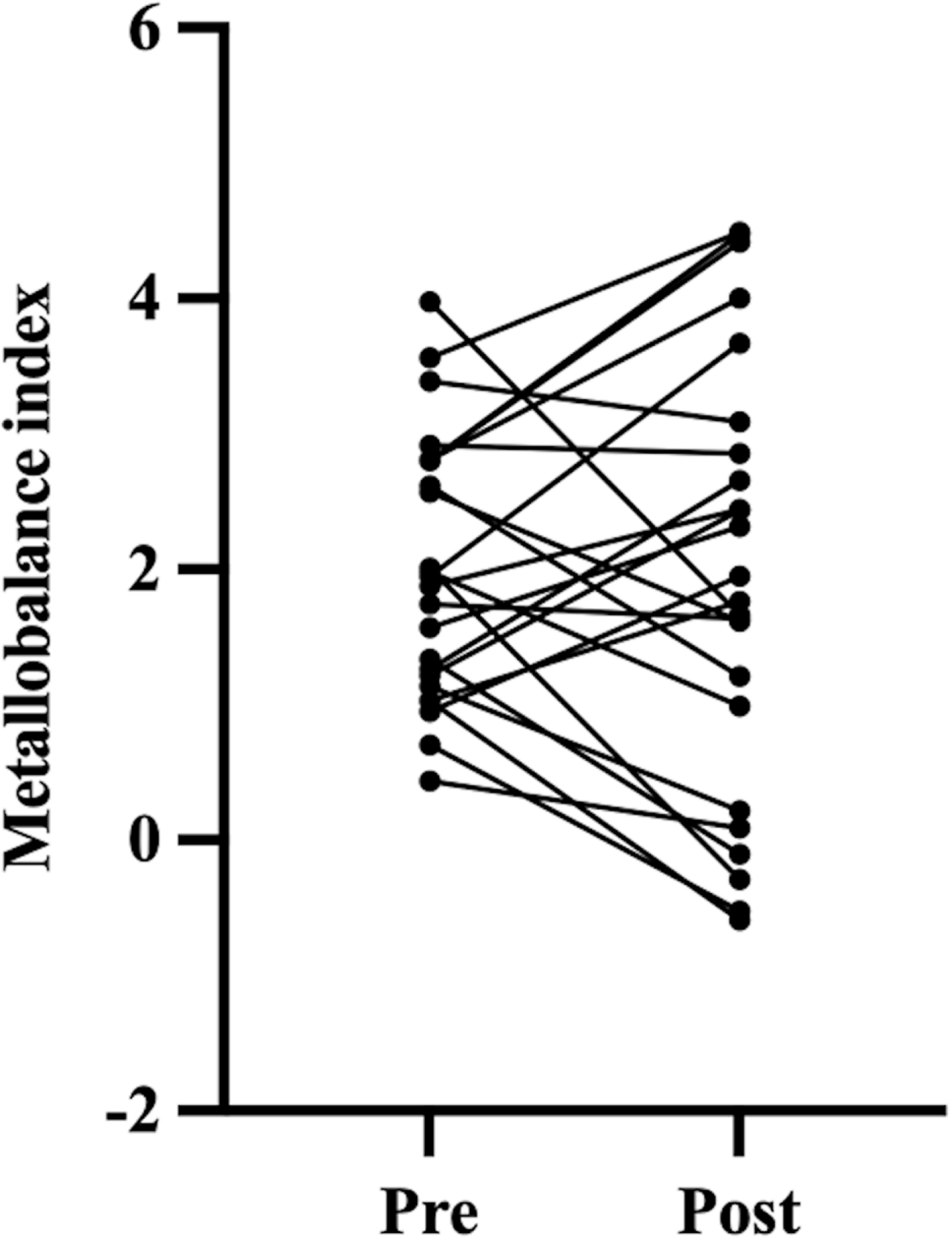
Paired Metallobalance index values before and 2 weeks after surgery in dogs with prostatic adenocarcinoma. No clear short-term change was observed between preoperative values (1.92 [1.16–2.80]) and postoperative values (1.86 [0.41–3.03]; *p* = 0.8774). The *p* value was obtained using the Wilcoxon signed-rank test, and the comparison was exploratory.

Preoperative MBI values were higher in dogs with lymph node metastasis (2.71 [1.93–3.18]) than in dogs without lymph node metastasis (1.45 [1.03–2.63]; *p* = 0.0330; Figure 7). Preoperative MBI values did not differ clearly between the R0 (2.71 [1.34–3.24]) and R1 (1.66 [1.10–2.43]) resection groups (*p* = 0.2381; Figure 8). In dogs with paired samples, MBI values did not show a clear short-term change between the preoperative measurement (1.92 [1.16–2.80]) and the measurement 2 weeks after surgery (1.86 [0.41–3.03]; *p* = 0.8774; Figure 9). These subgroup findings were exploratory.

## Discussion

This study established serum PRIs for 18 elements in clinically healthy male dogs and found that an 18-element LDA-based MBI showed high apparent discrimination between canine PAC and controls, including clinically healthy dogs and dogs with benign prostatic disease. The primary analysis yielded an AUC of 0.983, with sensitivity, specificity, and accuracy of 100.0, 92.3, and 93.9%, respectively, at the selected cutoff. These values are apparent training-set estimates because the model was developed and evaluated in the same dataset, and they should not be interpreted as validated clinical performance.

Reports systematically describing canine serum elemental PRIs remain scarce, and a previous study provided PRIs for 13 elements (9). The present study expanded the panel to 18 elements, and overlapping elements such as Cu, Fe, Mo, Se, Zn, and As were broadly comparable with previously published ranges. Broader applicability of the proposed PRIs will require standardization of sampling, storage, control of hemolysis and lipemia, digestion, and instrument conditions, together with verification across regions and institutions.

Canine prostatic neoplasia is often locally invasive and metastatic and is associated with poor prognosis, making earlier diagnosis clinically important (12–16). The MBI may eventually serve as a noninvasive adjunct to help prioritize advanced imaging, cytology, biopsy, or other definitive procedures in dogs with suspected prostatic disease. It should not be regarded as a replacement for clinical assessment, imaging, cytology or biopsy, and histopathological confirmation. BRAF mutation testing in urine DNA is a complementary molecular approach, but its sensitivity for PAC is limited (18, 19).

The similar apparent AUC observed after restricting controls to dogs aged 7 years or older reduces, but does not eliminate, concern about age confounding. Restriction did not create an individually matched design, and a nonsignificant age-comparison *p* value does not demonstrate equivalence. Residual confounding by age and other covariates therefore remains possible, and the focused analysis does not establish that the index is cancer-specific. Likewise, the high apparent AUC in the BPH-versus-PAC comparison is clinically encouraging but does not provide independent validation because both groups contributed to the original model-development dataset. In both analyses, the Study 1 equation was applied without refitting.

The MBI summarizes a multivariate elemental pattern and should not be interpreted as evidence that every included element changes in a uniform direction in every affected dog. Exploratory comparisons suggested differences in Na, Ca, Cu, Zn, and Se, partially overlapping with multi-element findings reported in human prostate cancer (6). Related human Metallobalance studies primarily support cancer-risk discrimination (6, 11); they do not establish prognostic or treatment-monitoring utility. In the present study, the absence of a clear association with surgical margin status or short-term postoperative change does not support use of the MBI as a validated prognostic marker or for 2-week postoperative monitoring.

Mechanistic interpretation is limited by the observational case-control design. Copper participates in metalloenzymes involved in angiogenesis and cellular metabolism (25). Selenium contributes to antioxidant and redox homeostasis through selenoproteins, and its associations with cancer biology appear context dependent (26–31). Calcium and zinc have also been linked to prostate cancer biology and systemic metabolic changes (32–36). The observed profile may therefore reflect tumor-related biology, systemic inflammation or metabolism, nutritional status, organ function, or other unmeasured factors rather than a single causal pathway.

The higher MBI in dogs with lymph node metastasis was exploratory and requires confirmation in larger cohorts with standardized staging and long-term outcome data. The lack of a clear short-term postoperative change may reflect the 2-week sampling interval, relatively stable systemic homeostatic alterations, residual disease, or limited statistical power. Prospective longitudinal studies should incorporate tumor burden, standardized staging, serial sampling, recurrence, and survival outcomes.

This study has several limitations. The numbers of clinically healthy dogs used for PRI derivation and dogs with PAC were modest. The retrospective, multicenter case-control design did not permit complete control of age, neuter status, breed, diet, supplementation, medication, and other covariates. Urinalysis was not performed uniformly in the clinically healthy cohort; consequently, subclinical renal or urinary tract disease could not be completely excluded and may have influenced elemental concentrations. Detailed information on mineral or vitamin supplements, therapeutic diets, and medications was not consistently available. Body weight and other detailed clinical variables were not consistently recorded, and histological tumor grade was not uniformly reported for PAC cases. Preanalytical and analytical variation, unadjusted exploratory comparisons, and small clinicopathological subgroups further limit inference. Most importantly, no internal or external validation was performed. Future work should prioritize prospectively collected multi-institutional cohorts, predefined covariates and sampling protocols, and independent validation. Larger datasets may also support evaluation of regularized or machine-learning models and direct comparison with the prespecified LDA-based index.

## Conclusion

ICP-MS-based multi-element profiling coupled with LDA showed high apparent training-set discrimination between canine PAC and controls. Focused analyses using older controls and the BPH subgroup reduced some clinical concerns but did not constitute independent validation. The exploratory association with lymph node metastasis warrants further study, whereas the present findings do not support short-term postoperative monitoring or a validated prognostic role. The proposed PRIs and MBI require independent prospective validation before clinical implementation.

## Data Availability

The raw data supporting the conclusions of this article will be made available by the authors, without undue reservation.
